# Learning journeys – student learning development in the first years of a medical degree: an analysis of student conversations

**DOI:** 10.3389/fsoc.2023.1244039

**Published:** 2023-12-04

**Authors:** Kerry G. Gilbert

**Affiliations:** School of Clinical and Biomedical Sciences, Faculty of Health and Life Sciences, University of Exeter, St Luke’s Campus, Exeter, United Kingdom

**Keywords:** learning journey, problem-based learning, medical education, narrative analysis, thematic analysis (TA), listening project, conversations about learning, understanding learning

## Abstract

**Introduction:**

Students starting medical school generally come from a learning background that expects them to learn content, which is reproduced to pass an exam. As a part of their learning development, they must adapt and become self-motivated learners who can determine the underlying principles or concepts and use these to problem solve in the uncertainty of real-life clinical practice. Whilst much has been written about designing curricula to promote learning development, there is no one-size fits all approach to facilitating this type of learning, thus an analysis of what helps and hinders learning development is indicated.

**Methods:**

Student pairs in Y2 and Y3 of an undergraduate Bachelor of Medicine, Bachelor of Surgery (BMBS) programme of a South-West UK medical school, were asked to audio record a conversation about their learning through a facilitated problem-based learning approach during the BMBS course so far. They were provided with a brief to aid them in their discussion in the style of the outside broadcast method of BBC Radio 4s listening project. Using this method, the conversation was unfacilitated and allowed to take its natural course. Conversations were transcribed and coded to determine emerging themes with respect to the developing understanding of the students about what and how they were learning.

**Results:**

Four student pairs volunteered for the project one from Y2 and three from Y3. Five key themes were identified including: from ‘learning it all, to structured learning’; ‘developing understanding and the spiral curriculum’; ‘working alone versus working with others’; ‘integrated learning and understanding context’ and ‘assessment and resources.’ Narrative analysis within these themes suggested that over the course of the first two to three years of study, participants developed a better understanding of how best to learn, although there were differences in both time and order that participants reached a point where learning felt more natural to them.

**Discussion:**

Analysis of the data suggested that students develop independently towards being self-motivated lifelong learners. There were several key aspects of curriculum design that could be used to facilitate this development, which could easily be incorporated into developing or creating problem- / enquiry-based curricula.

## Introduction

Students arriving on day one of the Bachelor of Medicine, Bachelor of Surgery (BMBS) undergraduate medical programme are predominantly school-leavers or school-leavers returning from a gap year. Secondary education in the UK still focusses on factual knowledge reproduced under exam conditions as a measure of success, rather than learning skills required to succeed in independent learning (Beers, 2006; Jones, 2011). Accordingly, to access the course, entrants must have been diligent in learning the syllabus of their A-levels. As a medical student and by inference a trainee doctor, it is not possible to know everything there is to know. Therefore, it is no longer enough to regurgitate knowledge and students must learn to remember useful key concepts that can be used to process and evaluate information in different situations. Students entering a medical (and many other university) programme(s) need to adapt quickly to a very different learning approach. This BMBS programme, presents an integrated curriculum across, multiple themes. Students in year 1 and 2 spend much of their time in the classroom learning theory and content through a range of learning activities including enquiry-based learning, lectures, interactive and practical classroom learning and small-group discussion. This is complemented by early clinical exposure through placements in a wide variety of healthcare settings, where they can see the value of their learning through working with patients. In year 3 and beyond, students spend more of their time in hospital placements, integrating and stimulating their academic learning through clinical exposure to a broad variety of patients on longer clinical pathways. Thus, it is necessary for students to adapt to a new learning environment early in the course. The curriculum design requires them to consider complexity and the broader perspective of medicine from the outset. Furthermore, assessment via a progress test that measures applied medical knowledge at the level of a newly qualified doctor demotes their attainment to single-figure percentages rather than the A/A* grades to which they have been used. This presents a dilemma for many students. Although they are marked against the rest of the cohort, in terms of their overall grade percentage, they are faced with a score that would previously, have represented failure, or at least, significantly below-average attainment (Rodway-Dyer, 2010). In addition, students are marked by their tutors in terms of their professionalism and ability to learn both individually and as a member of a team, as well as via more traditional means, such as essay writing and knowledge tests. Unsurprisingly, this presents a challenge to their learning approach, which for many includes a complete re-evaluation of learning from a syllabus to attain top scores, to development of understanding to progress. Within this, they must learn to weave in the knowledge that they learn from both traditional structured learning and personal experiences. Current thinking in terms of learning in any social setting, which by extension includes medicine, particularly where an holistic approach following the biopsychosocial model is employed (Engel, 1977), requires more than an accumulation of knowledge (McInery, 2018). Indeed, professional development requires a lifelong learning approach where students must start to transfer and use their knowledge to new or unfamiliar contexts. This in turn helps them to improve their understanding of each topic and develop their overall knowledge (Maton, 2009).

During the design and development of curricula, all stakeholders must be considered in deciding which concepts are necessary and relevant to overall understanding (Davenport et al., 2004). In an integrated medical education, this is complex. Multiple stakeholders with a broad-range of subject expertise, understanding of the complexity of human health and an appreciation of the bigger picture of healthcare delivery are needed. A particular difficulty arises from differing opinions between discipline experts from academic and clinical arenas as well as from the student cohort (Kobus, 2013; Quinlan et al., 2013). This is not exclusive to medicine, dissonance has also been highlighted between stakeholders in other disciplines, e.g., engineering education (Knight et al., 2014). Morcke et al. (2006) report the completely different perspectives of stakeholders, regarding what learning is required and the way in which it should be delivered. Thus, it is proposed that the cognitive aspects of learning and the ability to develop understanding alongside the socio-cultural construction within the learning environment play a key role. Whilst much has been written about curriculum design to promote useful learning skills, little has been reported on how students view the changing landscape of their learning. Specifically, there is little written about what students perceive as getting in the way of learning (barriers) or what helps them to take steps towards success in acquiring effective and lifelong learning skills (enablers). Therefore, the approach to optimise learning context, should be exploratory and reflective, as there is no single, one-size fits all formula to ‘crack’ learning in all contexts (Kobus, 2013; Barradell and Kennedy-Jones, 2015). This would suggest the need for a thorough analysis of what causes difficulty for students in the context of the learning environment and how various approaches can help or hinder the learning process as well as considering the opinions and approaches of discipline experts and educationalists (Noonan, 2013).

## Methods and methodology

### Using student-generated narrative

Narrative and stories are increasingly used in the health and the social sciences to better understand the lived experiences of people’s lives. There is no specific or predefined way to analyse the stories told, so interpretation relies on the individual perspective of the listener to make sense of the ‘message’ that comes from the (re)-telling of the participants lived experience (Lai, 2010). Stories can be told in different ways and the method of storytelling affects the ensuing narrative. Thus, narrative can be impacted by a number of factors, e.g., time of telling; audience; whether it is told as an individual monologue or unfolds as part of a conversation etc. (Charon, 2006). From this viewpoint, it can be surmised that narratives will also change over time, with re-telling and with the developing experience of the teller or, because of the influence of the listener, particularly if it is part of a conversation or discussion. Storytelling is a natural part of human behaviour, we all tell stories to justify our actions, reflect upon and learn from experiences, to continue our personal journey of development. The relationship between the storyteller and audience, is crucial to the telling of the story (Squire et al., 2008). If the storyteller feels that they will be judged by the listener, they are more likely to tell their story in a self-affirming and uncritical way, or even altered to protect the teller. To a varying extent, storytelling leads to self-reflection and helps the teller to search for advice, support or affirmation for their position (Bolton, 2006). Thus, storytelling can be adopted in qualitative research to unpick and seek to understand the issues being researched. In so doing there is a co-construction of understanding to make sense of the relevance of the lived experience. Narrative enquiry is a powerful tool in the development of meaning from experience to underpin improved understanding of a community or group (Berry, 2016). In terms of learning in a specific setting, this provides a useful tool to shape the environment to enhance the experience of individuals in a learning community. As the analysis of narrative is subjective by its nature, care must be taken to consider the form in which the data is collected and to limit the influence of the researcher in the telling of the story (Lai, 2010). The Listening Project encourages participants to discuss shared experience privately, in a radio booth, recorded for sharing on national radio (“Radio 4: The Listening Project”, 2016). Resultant conversations are influenced only by the pair having the discussion, as there is no set process or questions to lead their discussion. As a method for collecting data, this provides an opportunity to explore narrative away from the influence of the researcher responsible for, and thus influencing, the interpretation. This means that the influence of the researcher is confined to the interpretation of the already recounted story (to some extent), rather than during the data collection process (Maynes et al., 2008).

### Data collection

Students were recruited from Year 2 and 3 of the Bachelor of Medicine, Bachelor of Surgery (BMBS) Course via email, asking if they would like to take part. Participants volunteered as a pair and in this respect were self-selected, rather than being randomly assigned to a pair. This was intentional as it was felt that the students would have a pre-existing rapport, rather than needing to develop a relationship during the development of a conversation. This is in-keeping with the Listening Project from which this methodology was derived (“Radio 4: The Listening Project”, 2016). All participants were given pseudonyms to preserve anonymity (Table 1).

**Table 1:** Student pair labelling key: (f) = female participant; (m) = male participant.

Year group	Name 1	Name 2	Pair code
2	Annie (f)	Belinda (f)	A
3	Clare (f)	Dan (m)	B
3	Eashi (f)	Freddie (m)	C
3	Hannah (f)	Gafoor (m)	D

Each pair of students were given a brief explanation of the aims of the project in terms of understanding how students use group learning to develop their learning skills to cope with the volume of knowledge and understanding required to succeed on the course. Student participant pairs were then asked to record a conversation at a place and time of their choosing and given a conversation brief, outlining areas of interest of the project and some prompt questions to use if they got stuck (See below).

Conversation brief:

I am interested in hearing your learning stories (experiences), with this in mind, please consider the following questions:
Were your learning experiences what you expected? – How and in what ways were they similar or different?
What do you like about them? What do you dislike?
What has helped you to learn? What has hindered your learning?
Is there anything that could have been done to make your learning experience better?
If you were going to give some advice about learning, to your younger self starting this course, what would it be?

### Recruitment

Eight student participants were recruited as four pairs, two (one pair) from the second year and six (three pairs) from the third year of the BMBS course (Table 1). From this a total of 90.11 min of conversation were recorded comprising: 40.04 min, 8.55 min, 22.05 min, and 19.47 min for each conversation. Students used language indicating different stages of understanding in relation to being a competent learner. Interestingly, one of the Year 2 students and four of the Year 3 students appeared to have a deeper understanding of how their learning was taking shape as an organic and iterative process in which, understanding developed over time, whereas the other students seemed to struggle with how to build on what they had learnt and found learning much more a process of learning topics separately.

Transcribed conversations were read thoroughly several times and analysed according to similar narratives / themes arising *viz*: ‘from learning it all, to structured learning’; ‘developing understanding and the spiral curriculum’; ‘working alone vs. working with others’; ‘integrated learning and understanding context’ and ‘assessment and resources’. Quotations were selected to reflect these 5 main / key themes (see Table 2 for example).

**Table 2:** An example of theming of conversations.

No	Theme	Description
1	From learning it all, to structured learning	Participants discuss how at first, they felt that they needed to learn everything about medicine to succeed on the programme but came to understand that this was not the case and that learning was an individual experience.
2	Developing understanding and the spiral curriculum	Participants in all conversations mentioned the spiral curriculum. Whilst this was not a theme that dominated conversations it is key to understanding the learning process
3	Working alone versus working with others	Participants discussed their experiences of working in groups – particularly in terms of opportunities and missed opportunities
4	Integrated learning and understanding context	Participants discuss how they begin to understand the integrated nature of learning as they progress through the programme and have to apply their learning in a clinical context.
5	Assessment and resources	Assessment is discussed as a driver of learning early in the programme, as participants develop their learning skills it appears to become less of a driver.

### Analysis

Data was analysed using a mixture of thematic analysis (TA) to identify patterns and themes within the dataset in relation to the research question (Braun and Clarke, 2006) and narrative enquiry to consider the socio-cultural perspective of learning, rather than taking a cognitivist psychological viewpoint (Maynes et al., 2008). This approach facilitated the interpretation of practical barriers and enablers borne out of the student’s approach and beliefs about learning, rather than the cognitive process of assimilation of information and development of understanding (Case et al., 2010; Lai, 2010). The language used in the narratives obtained through listening project conversations were analysed to determine the course of participant journeys through struggle to understand how time, topic, sequence, student and facilitator interaction and context of learning empower or inhibit learning within the context of learning through the early years of this BMBS programme. Thus, as experience affects comprehension, language changes to reflect a deeper appreciation of the complexity and linkage of ideas that explain a more rounded understanding of a topic in the context of other issues or learning. In this context, this type of analysis focuses more attention on the process and development of learning, rather than the specific issues that students face at a particular moment in time (David and Sutton, 2011b).

### Participant use of the conversation brief

It was evident that some of the participants stuck more closely to the conversation brief (the brief) than others. Where conversation was freer, i.e., seemingly unhindered by the brief, there seems to have been a more iterative and developmental aspect to the discussion, with participants seemingly learning from their discussions, exploring ideas, and shaping their understanding as the conversation progressed. Interestingly, these conversations gave the impression that the researcher was ‘forgotten’ for some of the time. Whereas, participants who stuck more closely to the brief, appeared to have a less dynamic interaction, only answering the questions set by the brief. The conversations where the brief was more closely followed were in contrast to the freer conversations in that they showed much more evidence of researcher presence. Thus, it could be speculated that the methodology outlined above did, at least in part, ameliorate the effects of my influence collecting participant stories and led to a more personal recounting of the learning journey. It is also worthy of note that participants story-sharing led to changes in perception of their own understanding of learning. When this happened, the conversation veered into new areas of exploration, perhaps demonstrating a transformative effect of participants on one another, which could be ascribed to peer-to-peer learning.

### Analysis of themes to the data

#### From learning it all, to structured learning

Students come to medical school with the feeling that they are more than capable of learning, having spent many years getting the top grades at school and achieving A/A* grades at GCSE and A-level. Over the same time period, they have narrowed their learning topics and focused their study on individual subjects. A key aspect of success in this setting, is learning to a syllabus, taught directly by their subject teachers. This approach, whilst offering success in knowledge-based exams, stifles natural, spontaneous ability to learn organically through enquiry, discovery and thinking (Bonawitz et al., 2011).

Students are introduced to the PBL approach early in this BMBS programme and this was acknowledged by participants, as an issue for them as they made their transition to learning in the medical school environment, for example:

*I think this is quite a big jump [from 6th form, where] …there's a syllabus, there’s a beginning and an end*. (Gafoor C: Y3)

Medicine covers a broad range of topics, which crossing several disciplines and themes, not only biomedical science disciplines, but also arts, humanities and social sciences. Therefore, far from specialising, students diversify considerably to become individuals with a broad knowledge across a number of areas. This can be daunting and was reflected in participant conversations. There was a consensus early in the course that they need to learn everything they are taught:

*…there was so much information …such a short space of time and you really thought you had to know everything*. (Belinda A: Y2)

*I carried round a pack of about 100 maybe 200 …little questions with …if I knew all of these …I’d know I’d learnt everything*. (Annie A: Y2)

*…Year 1 was a lot of notetaking and …going over lectures …it was quite productive …I knew my stuff for end of year but …a long time was spent making notes*. (Clare B: Y3)

Flexner in the early 20th Century, suggested that pre-clinical learning should include a firm grounding in the biomedical sciences that underpin diagnostic medicine, before moving on to clinical training (Flexner, 1910). However, medical understanding has improved over the past 100-years and it is no longer necessary, appropriate or possible to learn everything, although this may not be appreciated by a student new to learning in higher education (Irby et al., 2010; Miller et al., 2010; Jones, 2011). Thus, the keystone of medical curriculum development is deciding the key concepts required to develop understanding and thinking pertinent to progression through the medical graduate foundation training programme, where new graduates are expected to determine their own learning development, whilst undertaking specific training to demonstrate capability as a practitioner in medicine (Norman, 2002; Mann, 2011). The medical curriculum adopted at this medical school attempts to equip students with the learning skills requisite for good clinical decision making, based on a firm grounding of the biomedical, psychological and social sciences (including current literature and evidence), alongside an appreciation of the holistic view of the patient (Bleakley and Brennan, 2011). Clinical reasoning sessions begin in Y3, when students have greater exposure to the clinical environment, but students in earlier years are introduced to the skills required to develop clinical reasoning thinking from the outset, through problem-based learning and other small-group sessions (Schmidt, 1983; Mattick and Knight, 2007; Artino, 2008), which draw on their learning throughout the course. This approach aims to ease the transition, which is often seen as problematic, from largely non-clinical learning in Year 1–2 to the experiential, self-directed experience encountered in the clinical pathway framework from Year 3 (Rodway-Dyer, 2010; Teunissen and Westerman, 2011).

As the participants began to discuss how their learning progressed, there was disagreement as some started to explain that their learning had become targeted, or ‘smarter’, instead of learning topics in detail, they were beginning to understand that some of the key underpinning concepts would facilitate their work with new material. This was juxtaposed with the apparent reluctance to let go of the feeling that if they did not know everything, they might miss out. In the early part of the conversation between Belinda and Annie (pair A). Annie expressed her frustration that at school she knew where she was, there was a syllabus, you were taught, you were tested on what you were taught, and you passed the exams. It was clear cut, there was a pre-determined and definite path to success. Annie also commented about not being tested on Year 2 learning, there is no end of year test as there was in Year 1, which made learning even more difficult. She felt that it was no longer necessary to attend any teaching sessions, because there would be no test. Whereas Belinda found that she enjoyed the new freedom to follow her curiosity and was not encumbered by a syllabus, having scope to learn as she saw fit:

*I liked the way we were taught in secondary school was sit down, shut up, this what you need to learn… …get tested on the stuff that you’ve actually learnt.* (Annie A: Y2)

*…it’s all chill …I understand it, whereas you …you’re actually …this is way too much and stressing out.* (Belinda A: Y2)

Annie and Belinda’s discussion also highlights the feeling of some participants, that everything needs to be taught and that their lecturers are experts who can impart their wisdom, for their absorption, so they too can become ‘wise’ (Wingate, 2007). This is commonly experienced across higher education, presumably because of the syllabus-led school system (Teunissen and Westerman, 2011), rather than an expectation that higher level learning requires a more active participation and curiosity to develop understanding (Magolda, 2001; Bassendowski and Petrucka, 2013).

Interestingly, there seems to be a hint that Belinda (pair A) experienced a different approach in school, which has potentially, allowed her to adapt more quickly, affirming the notion that transition is influenced by expectation and prior experience (Byrne and Flood, 2005; Teunissen and Westerman, 2011):

*… they’d explain it and you’d understand it… …you’d talk about it and talk to the teacher and it was so much more open relationship*. (Belinda A: Y2, discussing how her 6th form teachers approached learning)

As conversations developed it was interesting that some students started to change their perception of how they might learn. For instance, as one of the pairs was talking, it became clear that one participant was struck by the difference in their approach compared to their research partner’s and even appeared to have a change of perception on how learning might be made more effective. Considering the developing notion that learning does not need to be structured in terms of specific learning outcomes, but rather in development of understanding of the broader topic, with an emphasis on the concepts that underpin current medical knowledge, Annie and Belinda (pair A) exchanged ideas that provoke thinking about what is holding Annie back. Annie is clearly worried that by opening the box, she will quickly be drowned by too much content, whereas Belinda seems to be confident that by following her curiosity she can learn enough to develop critical thinking, which she can use to work with ideas to synthesise new understanding:

Annie: *I just don’t get on with the whole, like go away, go free …there’s nothing that I’d particularly go ‘that’s really interesting’ …and research more into …I don’t like the freedom of it basically*.

Belinda: *I feel like the opposite …I’m not very good at structured learning …I like to follow my curiosity*.

*…last year I thought I knew what I was doing [but] …I figured out a new way of learning and I think I’ve learnt the mentality of work smart not hard …I think that it’s definitely a journey*.

Belinda’s vociferation that it is about working smart; considering what she is learning and how it fits in, echoes a development of understanding of the learning process and fits with developing evidence that deep learning is underpinned by individual goal-setting, rather than by following pre-determined learning outcomes (Mattick and Knight, 2007; Wijnen et al., 2017). This is a common theme in year three participant conversations, as they appeared to be more in-tune with the notion that their learning needed to be more holistic, rather than focussed on the knowing of facts. This is epitomised in Freddie’s (pair C) comment below:

*…I feel like if you did it in the didactic compartmentalized way that they’re doing in a lot of other medical schools you don’t have the ability to form those connections* (Freddie C: Y3)

By Year 3 Eashi is beginning to gain an appreciation of what is expected and seems able to reflect on the issues that faced her as a first-year student. As someone who had spent time on an undergraduate biomedical degree course, before joining the medical programme she discusses her confusion at trying to learn holistically, rather than in the discipline-led style she had encountered previously:

*…having come from the biomed route …looking at things …in separate courses …in real life you like you have a patient it’s not a separate thing …all of that stuff is happening in one person, …from an undergraduate perspective it’s really kind of a bit confusing* (Eashi C: Y3)

In considering the transition from a largely didactic approach to teaching, to the more experiential, guided approach, it is interesting to note that by Year 3, students are largely more accepting of the process and appreciate the benefits. This raises the question of whether earlier intervention to develop this way of learning could or should be included. Eashi sums up this progression:

*…first years and second years …are like criticizing it and complaining …when I hear about 3rd, 4th and 5th years, most of them are singing the praises of their learning and learning style of Peninsula and the spiral curriculum …but it takes that hindsight to actually see the worth.* (Eashi C: Y3)

The experiential nature of this transition, is perhaps, a first step to adaptability in the professional clinical environment and may begin to explain the feeling by students that they are prepared for practice on day one of their first Foundation Year job (Goldacre et al., 2010; Bleakley and Brennan, 2011; Illing et al., 2013). The comments above reflect that Freddie and Eashi realise that their developing understanding of whole issues, which alongside their comments below further supports the idea that they feel as if they are developing the required skills for clinical practice:

*I actually feel like I’m becoming a doctor rather than – I know about physiology or I know about the kidneys like I know about being a doctor of the kidneys*. (Freddie C: Y3)

*…it’s made me feel more confident with my clinical reasoning because I talk about things I’ve looked up rather than just reading it off a piece of paper*. (Eashi C: Y3)

The development of a professional identity is paramount to the success of students on an undergraduate medical programme. It is common in traditional undergraduate medical programmes, for students to start to grapple with this as they enter the clinical years. From this study, it would appear that our participants have already begun to develop their personal identity prior to Year 3 when, traditionally the clinical years, and ‘learning’ professional behaviour begins (Arnold et al., 2005; Clandinin and Cave, 2008; Hatem and Halpin, 2019). This is in-keeping with this school’s approach, where students are exposed to clinical experiences early and are provided with learning spaces, such as small-group learning and problem / enquiry-based learning to practice their professionalism from early in the programme. Indeed professionalism is judged as part of the assessment process by their academic and clinical facilitators throughout the course. The literature on transition would seem to support this notion, particularly easing the transition by encouraging earlier introduction of experiential and curiosity-based learning (Fallows and Steven, 2013).

Participants coming from school, where all they need to learn is taught by a teacher, expect higher education to offer the same. Thus, the anticipation that they will be taught enough to pass the course is implicit in their understanding (Wingate, 2007).

Interestingly, Annie suggests that a GP would not need to know the details of Kreb’s cycle:

*There’s no way a single GP knows the Kreb’s cycle…* (Annie A, Y2)

Annie expresses a view that learning is there to be done, but then forgotten, and so ‘what is the point?’ This echoes how I felt as an undergraduate in biochemistry. What was the point of learning the whole thing just to write it down in an exam? This would appear logical, but by understanding the mechanism of the pathway, the knowledge can be applied. Knowing what it does and how, helps when seeing patients, so, in a sense, Annie is right, GPs are unlikely to remember the detail of Kreb’s cycle ‘in the moment’, but they will understand the underlying principle that it is a generator and producer of energy and that if it is not functioning effectively, it will cause particular issues for a patient. Furthermore, they will be able to apply their understanding of the biochemistry, to other disorders of metabolism.

Thus, although the detail is ‘forgotten’ understanding of the system has, to some extent, become implicit. Should the GP need to know more detail, they will find it [relatively] easily and apply it to their case. Annie’s next comment hints that she is starting to get the point, an oscillation between thinking it is not necessary to learn only to forget, but then beginning to realise that the learning and forgetting aspect of understanding is fundamental to development of understanding required as an experienced practitioner:

*I understand we have to know all the nitty gritty detail now, …so that we can forget it later, but we have the basis for it.* (Annie A: Y2)

But then…

…*PBL catches the extra things, which you maybe should have learnt that week, but you just haven’t, they haven’t been taught to you properly* (Annie A: Y2)

Annie then flips back to her original position that the details need to be taught ‘properly’, although acknowledging the role of PBL in development of understanding in context, she sees it as a safety net, rather than a means to develop her comprehension. This oscillation between knowing what needs to be done and how to do it in terms of learning, measured against the feeling of wanting to be taught is interesting. Later in the course, there is still an acknowledgement of the difficulties faced earlier on, but an acceptance that learning is a journey, requiring personal development. This reflects the oscillation described by threshold concept literature, where there is a period of understanding and not understanding before a permanent transformation to the irreversible state of ‘getting it’ (Meyer and Land, 2003; Perkins, 2006).

#### Developing understanding and the spiral curriculum

During induction when the students arrive in Year 1, the BMBS course is presented as operating a spiral curriculum (Harden and Stamper, 1999; Rodway-Dyer, 2010), meaning that each area of learning will be presented at various times as the course progresses. Thus, by learning the basic concepts in the first year, students will lay a foundation, which provides a basis for future learning. This does not mean that each topic is taught to a greater depth each year, but that general concepts embedded in the core curriculum are used in new contexts and situations to develop a thorough understanding, allowing transfer of knowledge and engendering the skill to work with new and unfamiliar issues in clinical practice, using prior learning (Mattick and Knight, 2007; Rodway-Dyer, 2010). This approach is key to developing understanding and although it represents a small proportion of participant conversations, it is a key aspect of learning development and the timescale and process by which it happens is worthy of note.

Although the spiral curriculum and its underpinning theory, is introduced at the start of Year 1, there appears to be an urgency amongst Year 1 students to learn everything in as much detail as possible, as demonstrated by Belinda’s (pair A) reflection:

*Last year I felt very much like a lot of stress because there was so much information and such a short space of time …you really thought you had to know everything* (Belinda A: Y2)

However, by the middle of Year 2, there appears to have been a shift in these participants’ perception of how the curriculum works, which aids in organising workload and trusting that learning is iterative in its development over the undergraduate years. Indeed, by mid-Year 3, the participants in this project were convinced by the value of the spiral curriculum and really appreciated what it had to offer. Reflection on the value of the spiral curriculum is echoed in comments by three of the participant pairs, two explicitly and one by implication, for example:

*…repetition is really so important, …the spiral curriculum …you know it’s gonna come up again and every time it comes up you get sort of like a new piece of the jigsaw* (Belinda A: Y2)

*…thinking about other medical schools …they do …one case unit on the heart in the entire two years. You’re never gonna look at the heart again, I’d dread that* (Annie A: Y2)

*…that spiral learning, which when you start seems a bit like …so we're just going to revisit things again and again it's a bit frustrating …but actually you …just build on that knowledge and put another layer* (Eashi C: Y3)

*Don't get too hung up on …the minute details of an ECG for example. …you're not going to learn it unless you see it and you have at least three years …What you need to do for first and second year is to learn basic science* (Hannah D: Y3)

The comments participants make about the spiral curriculum and how their appreciation of it as a concept develops over the first few years, would suggest that in this case, learning for themselves is an important aspect of learning development, which is in-keeping with the findings of an earlier review of this curriculum (Mattick and Knight, 2007). This work emphasises that although the evidence is presented early, it is not until students experience it in practice and understand the advantage it imparts, that they begin to accept it as a useful learning tool in curriculum design (Mattick and Knight, 2007), which would seem to be borne out by the conversations recorded as part of this study.

#### Working alone vs. working with others

A key element of working as a healthcare professional, is working collaboratively in teams. Patient care is regularly discussed in multi-disciplinary teams (MDT), thus learning to communicate and work together is key to becoming a doctor (General Medical Council, 2009). Educational research also presents a plethora of evidence to support learning as a team is effective (Levin, 2005; Madrid et al., 2007; Stankov et al., 2012; Thondhlana and Belluigi, 2014). Interestingly, participants described learning as a group as an alien concept and often struggled to understand how it might benefit them:

*I personally like working in my room by myself doing my own thing at my own pace…* (Annie A: Y2)

*I never liked group learning and never got it, cos I just thought like being with other people was such a distraction* (Clare B: Y3)

*I was just like reading through the power points …going over and over them again, I did actually make quite a lot of notes …I had a folder full of notes* (Dan B: Y3)

On the other hand, others recognised group learning as an advantage from the start:

*…one of the things that attracted me to Plymouth …the whole group-based aspect of it, being allowed to discuss stuff. I always found it really helpful.* (Hannah D: Y3)

*I know for me that I'm a discussion-based learner, I learn by chatting to people …thinking of it out loud and talking myself to an answer …it will click in my head* (Freddie C: Y3)

*…I will talk through it and I might say a bunch of things that might be totally irrelevant …I'm making links to all the previous learning …which actually you do a lot and you don't even realise* (Eashi C: Y3)

An important consideration for people working as a group is how much each person gains as an individual. It is commonly expressed that the group’s overall learning can be detrimentally affected by students’ who do not ‘pull their weight’ (Thondhlana and Belluigi, 2014). If a student has experienced this previously, or within group work in PBL, it is likely that their opinion of group work as a means for learning will be negatively impacted. However, if they have good, or improving experiences, their opinion of how working with others benefits them develops over time. By incorporating group work through PBL and small-group sessions from the outset, students are strongly encouraged to develop their group-working skills. Indeed, as group-working is included as part of their professional assessment, it is incumbent on them to work effectively together. For students who have been encouraged to discuss and learn together previously, e.g., Belinda and Hannah – they can see how their learning has developed using this approach, whereas those who have started from a more traditional learn and regurgitate, e.g., Annie and Dan (pair A and B respectively) – seem to take longer to appreciate the advantage of group learning.

Freddie reflects that some groups were better than others and that the success of the group is based on who is involved:

*…when you put 10 people together, you’re going to have different personalities and also different learning styles* (Freddie C: Y3)

Listening to participant conversations, I heard a lack of motivation to work with other students, within their friendship groups. Thus, whilst they were beginning to appreciate the relative benefits of working as a team, there was not a concurrent development of understanding that made them think beyond their course-imposed groups:

*My housemates obviously all do medicine and there’s seven of us in total, …that’s a resource we don’t really tap into that much…* (Belinda A: Y2)

Belinda goes on, as she starts to realise the missed opportunity, to consider why this might be the case:

*… [we’re] all really good friends, all relate to each other and yet so many times people I know, friends …don’t feel that they can share weaknesses with each other …we very rarely come together, actually study together.* (Belinda A: Y2)

Interestingly, Belinda seems to suggest that it is an element of competition that causes students to shy away from being collaborative with their friends. This is interesting, as it has been reported that students in a competitive environment are less likely to be collaborative (Butler and Kedar, 1990). However, as students are encouraged to collaborate from Year 1 it might reasonably be assumed to be sending a message that collaboration is a key skill for learning. From this it could be postulated that the perceived competitive nature of assessment has a negative impact on student learning development early in the course, where it could be advantageous for students to collaborate to develop self-direction of their learning. As the course progresses, this seems to become less of an issue. In Year 3, participants have moved into a clinical setting where team-working is an expectation. At this stage of the course, participants have less formal group time with their peers and more ward-based learning. Thus, it would seem their appreciation of peer-learning heightens as the structured opportunities for it diminish.

Freddie (pair C) voices the dissonance raised in some participant conversations. Explaining that he can now see why groups are changed, although at the time it felt uncomfortable. This suggests that it is only by experiencing group change in the clinical environment and how it alters dynamic, that they become aware of the educational value of group changes earlier in the course:

*…you have to adjust your learning style, which I can see why they mix it up because obviously in real life …we have MDTs …and you have to learn to work with other people …[but] it can throw you off kilter a little bit* (Freddie C: Y3)

Eashi reinforces this in response, explaining that she now finds it easier to adjust when she moves from one team to another:

*…you can adjust to that style of then being thrown around because you know that you're more confident in your own abilities, …for a while you just feel a bit lost* (Eashi C: Y3)

Gafoor displays confidence that his learning has effectively prepared him for learning within the clinical environment:

*…now being in my clinical years I can really see sort of the impact of studying in a PBL way. I feel quite prepared to enter the clinical environment.* (Gafoor D: Y3)

Whilst it is difficult to tell whether by this point in the course, students have learnt to share ideas and develop learning, or whether inculcation in a clinical environment, where team-working is necessary for the provision of effective patient care, is difficult to tease apart, given that there is no control group on which to test the hypothesis. However, evidence gathered on preparedness to practice drawn from students (Monrouxe et al., 2009; Brennan et al., 2010) and from their supervising clinicians (Morrow et al., 2012), would suggest that early group work plays a role for the high scores achieved by this school.

#### Integrated learning and understanding context

As discussed earlier, participants described how they tried to learn everything that they came across in the formal ‘taught’ part of the course. This was particularly evident when participants discussed their learning during the first year:

*…anatomy wise, I used to just print out diagrams and try and remember. I don’t think that was the best at all to learn anatomy.* (Clare B: Y3)

Clare qualifies this by explaining the lack of context as they started learning. So, although there is an acknowledgement that trying to learn by rote may not be the most effective means to learning, without experience of other content / context, it is difficult to apply a different technique. Mattick and Knight (2007) and Finn et al. (2010) suggested that adding a context stimulus to learning can affect information recall, and Clare’s comments seem to reflect a feeling that this is the case in the context of learning early in the BMBS programme, where clinical experience and thus context on which to pin learning is limited (Mattick and Knight, 2007; Finn et al., 2010):

*…but then what else were we supposed to do because we’ve not like got much patient contact or anything* (Clare B: Y3)

Dan (also suggests that lack of context made learning, particularly anatomy, quite difficult):

*That was always a great problem with anatomy, it was just like we were remembering stuff with no context* (Dan B: Y3).

For most participants, it becomes evident that their learning approach shifted as they progressed, so instead of only learning topics they had ‘covered’, they began to cross-reference to the work they had done before, e.g.:

*…I get all the questions that I’ve had from last year and I’ll make notes …by the end of it I’ll have had one set of notes …I’ve kind of paired them up with the case units from last year*, (Annie A: Y2)

There was a general impression that the participants who had progressed into the clinical environment appreciated the approach to teaching and their learning development, in terms of how it helped them to adapt to the clinical environment. Freddie sums this up, explaining that the PBL approach, in terms of setting his own learning goals, means he is more ready to learn from clinical experience and more confident of uncertainty as an acceptable part of clinical competence (Simpkin and Schwartzstein, 2016):

*…if someone just told me a bunch of learning points …I wouldn't have developed the skill that we need in clinical practice which is: what don't I know, what uncertainties are there and what's the information I'm lacking* (Freddie C: Y3)

Freddie’s comments suggest that he is becoming adept at lifelong learning skills. He can assess a situation he has not previously encountered and work out what he knows in order to manage the situation and what he does not know and will need to learn or ask someone for help, depending on the urgency and context of the situation. This is a key professional skill for doctors – knowing what you know and can deal with and what you do not know and need further knowledge or help with is crucial for patient safety (Passi et al., 2010; Monrouxe et al., 2011). As a Year 3 student, that Freddie is, at least, beginning to be able to assess his own strengths and weaknesses, is a big step towards developing his professionalism and ability to operate in the clinical environment (Goldacre et al., 2010; Illing et al., 2013).

When working with students in Year 1 and 2, Hannah recognises the change in her approach, in that she was now looking for clinical context, rather than learning for the sake of learning. This would suggest that the context of learning is an important factor and that it is difficult to understand what is expected in terms of learning without an authentic framework (Teunissen and Westerman, 2011; Teunissen et al., 2018):

*I think one of the difficulties with first and second [year] though was you never see the bigger picture because you're not in hospitals or placement enough.*

*…I was mentoring …year two students on how to do ISCEs …it was really interesting how my perspective was very different to theirs. …for them it's very much like I've got to say this this… …[whereas] I'm thinking ‘well what does this mean?’, ‘and how could I apply …biosciences that I've learned and all the pathology’ …it's very different now. …you put everything together don't you* (Hannah D: Y3)

It would seem from these observations, that student learning in Years 1 and 2, whilst abstract in terms of authentic clinical context, the methods of learning instilled in them are effective in aiding transition from non-clinical environment to the pathways of clinical learning introduced in Year 3. Working in small groups seems to be a factor in preparing students for the clinical world, where they are regularly moving between teams and forced to direct their own learning through understanding where the ‘holes’ or weaknesses in learning lie. This fits well with the observations of Teunissen and Westerman (2011), who suggest that medical education should be designed to help students cope with the challenges faced when entering new environments. Participants went further, articulating that as the course progressed, they felt like they were learning to think. This is a key reflection, as it highlights a complete transition in learning. So, learning is not about knowing, but being able to work with information. Eashi (pair C) makes an explicit comment about a sea-change in her learning:

*…you actually had to actually learn to think… …to look at different sources and find different information and apply knowledge to answer your questions* (Eashi C: Y3)

This seems something of a revelation to Eashi, which is perhaps surprising in the sense that, as a lifelong learner, it seems obvious that thinking is a key skill in development. It is interesting to note that children in nursery and primary schools are more skilled at enquiry-based learning than students starting medical school (Rogoff and Matusov, 1996; Cook et al., 2011). This is widely reported in the literature, although medical schools that use small-group or discussion-based learning such as PBL, report that their students are better problem solvers (Albanese and Mitchell, 1993; Norman and Schmidt, 2000; Koh et al., 2008). Thus, it is reasonable to assume that the ‘correct’ answer approach to examinations may slow the development to lifelong learning through the secondary school years. Indeed, this transition may be prolonged by continuing with a didactic approach in lecture-based courses, which does not appear to be lost on Eashi:

*…if you did it in the didactic compartmentalized way that they’re do in a lot of other medical schools you don't have the ability to form those connections …everything sits in its box* (Eashi C: Y3)

A key component in developing critical thinking is identified by Eashi and by Freddie (pair C) as they discuss how using questions to stimulate learning helped them not only to find information, but also how it helped with long-term memory and contextual understanding:

*…so questioning side of things …it helps you understand where to where to find things* (Eashi C: Y3)

*…it was surprisingly effective at making me remember stuff …and apply learning …in a more integrated way …I suppose it reminded me how to work with groups as well* (Freddie C: Y3)

Freddie goes on to discuss the development of a more holistic view of patients, suggesting the integrated approach to learning has helped him to consider patients as people, rather than just a presenting complaint or condition:

*…PBL teaches us to integrate the kind of psychosocial elements as well. I know we all think at the start what a load of rubbish, but then really when you talk to a patient all things are so important, …when you talk systematically and compartmentalised you don't really apply that stuff* (Freddie C: Y3)

This is also reflected in Eashi’s comments, where she explains how the early small group learning she has done has made her more attentive to the broader issues in patient care. Particularly, she discusses the detail that she is likely to consider, alongside the patient’s main issues:

*… it’s made me …more attentive to detail when patients talk, …rather than just thinking right here's the presenting complaint …the past medical history, done it's taught me to kind of think more holistically* (Eashi C: Y3)

Freddie appears to recognise that the integrated approach to learning, using a biopsychosocial framework, is crucial to consideration of the patient in terms of working towards wellbeing. He goes on to reflect on how this approach has helped him to make sense of discussions during multi-disciplinary team meetings (MDTs), on clinical placements:

*…I feel like even if I don't know everything that's going on I can pick up on bits and …notice things and I'm able to listen to a conversation that an MDT might be having and just make those links …it takes time though, it doesn't happen after your first PBL session* (Freddie C: Y3)

Freddie continues to discuss a PBL case he remembers that provided him with a framework for asking pertinent questions and considering the available services to help the patient:

*…there was one [PBL case] about carers and it was …how can we make sure that the carers are doing ok, support groups for carers …all that psychosocial stuff, it's not …just like a tick box thing it really has a lot of implication on someone's recovery and health* (Freddie C: Y3)

He goes on to relate this directly to a clinical experience, where the issues considered in the PBL case can be used to help understand a patient’s needs. This example clearly demonstrates his ability to use the learning from PBL, in a real clinical scenario; not as a learnt behaviour, but as a cognitive process, where what has been learned is being actively and deliberately applied to a real-life clinical case. Thus, the premise that PBL is a valuable learning tool for the development of critical thinking and clinical reasoning is demonstrated in Freddie’s lived experience (Prince et al., 2005):

*…if someone's a bit down… a bit worried about the fact that they're not going to be able to …walk after their knee or hip replacement.*

*…[if you] link that into their [mindset] and then they might be more positive… …not just like this is how you do the surgery, it’s understanding …about their recovery, the post op care …all that stuff …I wouldn't necessarily always think about but having …PBL …helps you think, look at the bigger picture.* (Freddie C: Y3)

Freddie’s thoughts provoke Eashi to reinforce his comments, as she also recognises how her learning in PBL has helped her to consider the whole patient, rather than just dealing with their immediate medical concerns:

*That's just exactly what I was just thinking …makes you remember that patients have a life outside of the hospital …you're dealing with an individual case but there's so much surrounding this case, …all their home environment …family, …children, …friends, …work and you remember …this isn't just an illness this is a person* (Eashi C: Y3).

This is a powerful affirmation that learning that has taken place over the first 2 years has allowed participants to enter the clinical world with a firm educational background, not just in the application of facts about medical conditions, but in considering all the patients’ needs to promote overall well-being. This would suggest that the journey towards lifelong learning has a firm basis in the early part of this programme. Furthermore, Eashi’s insight with respect to this understanding is cemented by the conversation she is sharing with Freddie, as she recognises their shared experience.

#### Assessment and resources

It is widely acknowledged that assessment is a key driver of learning (Epstein, 2007; Wormald et al., 2009). The progress test, an Applied Medical Knowledge test (AMK), is designed as a tool to monitor and assess progress frequently over the duration of the course (Ricketts et al., 2010). The test is set at the expected cognitive ability and understanding of F1 doctors, i.e., at a point where the candidates will have completed five-years of undergraduate training in medicine. Thus, students with very little or no training in medicine are bound to struggle with the content presented and their scores will be low. However, students will see a steady climb in score, which will show them how they are progressing in terms of their development as a doctor. The initial intention in designing this as a means of assessment was to provide a snapshot of how each student was progressing as they moved through the course, using their earlier scores as a reference point (McHarg et al., 2005). For adult / lifelong learners, this is a logical way to ensure appropriate development, so that any issues in learning development can be picked up early and remediated for to avoid short-term problems, becoming long-term issues. The original premise was that the test could be imposed at a moment’s notice, as it was not intended to test knowledge *per se*, but to visualise the development of students to apply their understanding and knowledge to clinical situations, using critical thinking and clinical reasoning skills (Van der Vleuten et al., 1996; Norman, 2002). This works well as students gain clinical experiences and opportunities to apply their learning to real-life cases, using their knowledge and understanding. However, it is often noticed that students in the early years of the course, who have little clinical exposure, lack the skills to apply clinical reasoning to complex scenarios and thus try to learn facts to attain higher scores. This is not surprising, given that as school-leavers they have come from a setting where attainment is measured by examination of factual knowledge. To ameliorate for this, Year 1 students have an end of year test that examines the learning they should have done during the first year. However, students in Year 1 and Year 2 often attempt to improve their AMK scores by learning clinical facts, e.g., disease frameworks and drug profiles. This may divert them from core learning, which in turn may be counter-productive in terms of longer-term progression on the AMK (Mattick and Knight, 2007). The effect of learning for AMK and the later realisation (or not) that this may be counter-productive was discussed in all the participant conversations. For example, Gafoor (pair D) points out that learning for tests is done without clinical understanding, because students are ‘intimidated’ by AMK. He does not say whether he did this in Years 1 and 2, but he does recognise as a third year that younger students over-emphasise learning for AMK:

*I think a particular reason …especially first and second years …seem quite hung up on the clinical context is, …AMK is quite an intimidating exam …a lot of students tend to be using all of these question-generating websites …learning buzzwords …looking at guidelines …managements for conditions that they don't really …know…* (Gafoor D: Y3)

Hannah (pair D) also suggests that this approach is counter-productive:

*…get rid of this culture …of learning for the AMK …it's a huge culture, particularly as you go into second year when you haven't got any other exams …The danger is you learn the basics, but you don't know the detail. So, you still get things wrong…* (Hannah D: Y3)

The issue is raised by Belinda, who seems to intonate that students somehow ‘cheat the system’ and improve their grades by learning for AMK. It appears that she recognises that students are not clinically competent, but by learning key facts and words, which lead them to the correct answers, they improve their scores, at least in the short-term:

*…I think actually, I find you can pass the exams and do quite well without necessarily having that clinically applicable stuff sometimes.* (Belinda A: Y2)

Furthermore, it is suggested that student re-runs of exams, explaining answers, without really understanding their clinical relevance or application is an issue in terms of learning in Years 1 and 2:

*…you’ve got some people to re-run exams who perhaps have no idea clinically… their actual knowledge of applying themselves to a medical scenario is really awful.* (Belinda A: Y2)

Annie seems to have a moment of realisation in response to Belinda’s comment, explaining that she had not seen the AMK in that way. She understands that it is clinical but had not thought that by learning and applying knowledge, she might achieve a better outcome in the exam over time:

*I’ve never thought of AMK like that, that has completely changed my thoughts …yeah AMK is very, very clinical.* (Annie A: Y2)

This is particularly interesting in terms of Annie learning from the discussion, as earlier in the conversation she had expressed her frustration that the teaching in Year 2 was not being tested, as there was no end of year exam.

A conversation between Clare and Dan (pair B), suggests that AMK became their sole focus in Year 2, to the detriment of engagement in learning in other parts of the course. This is put down to there being nothing else to learn for as there is no end of Year 2 test:

*…I think for Year 2 …the focus was just AMK, I didn’t do as many notes in Year 2* (Clare B: Y3)

*…even with PBL it fell off a bit towards like halfway through Year 2, I suppose. Most of it was not relevant …to the AMK, so I would just do AMK separately* (Dan B: Y3)

Gafoor and Hannah’s (pair D) conversation also makes this conclusion. The stage of learning in Year 2, is not enough for students to understand that their learning contributes to their progression in AMK and that, in their opinion, further testing would be an advantage in terms of encouraging learning for the content presented in Year 2:

*I think the reason perhaps people's attention to PBL in second year kind of changes, …it didn't really aid assessment. …we didn't have an exam to …consolidate our anatomy …physiology and pharmacology …apart from the AMK.* (Gafoor D: Y3)

He goes on to suggest that further assessment of the learning in Year 2 might lead to better engagement with learning content rather than superficial learning for the AMK:

…*different people would be more engaged with the content and be more likely to revise it and retain the knowledge. …that's just my view, …there should be more assessments.* (Gafoor D: Y3)

This is interesting, and fully reinforces that in the early development towards lifelong learning, students are driven to learn for assessments, rather than to work with the issues they face as part of their clinical experience. There are several ways of looking at this; it could be assumed that by increasing the number of assessments in Year 2, students would indeed pay more attention to the topics explored. From appreciation of the participant conversations discussed, it is likely that this would lead to a greater focus on taught aspects of the course, as there would be an immediate reward (i.e., a high grade) for learning the content in depth. This might seem at first appealing, but it could be argued that this would stifle learning development, as students would focus their attention on a narrow field of content, rather than being driven by the outward focus of clinical experience. In the early years of the medical school, students sat an end of Year 2 exam, which provided an alternative means to passing the Medical Knowledge module (*via*the AMK). Analysis of second year results, over a number of years, suggested there was no advantage to learning and the test was dropped.

In general, by Year 3 most students are taking AMK in their stride and seem to understand the testing of learning progress. In contrast, it is striking that if this is not the case – i.e., a student is still putting store in learning specifically for AMK that it is easy to negatively affect confidence in student learning. Dan’s (pair B) comment suggests he has not yet developed a learning to understand approach:

[About AMK] *…it only like takes small changes to make a huge difference and knock you out of your comfort zone so much.* (Dan B: Y3)

From this, it seems that the point at which assessment is no longer seen as a driver of learning, but merely a means of testing progress is not clear cut. However, Dan does appear to be alone amongst participants in this study, given Clare’s response:

*I feel like I’ve learned loads in third year, like tons and tons and tons of stuff. Lots of new things*. (Clare B: Y3)

Whilst she does not explicitly acknowledge her transition, her comment suggests that learning is more experiential, and curiosity driven than it was in Year 2.

## Discussion

Using the themes emerging from the student discussions as described above I will take each in turn and discuss how these fit with the curriculum at this medical school and with the more general topic of learning development with respect to progressing towards becoming a lifelong learner.

### Individual learning development from Year 1 to 3

The evidence presented here suggests that the transition from school leaver to medical student entering the clinical environment in Year 3 is helped and / or hindered in a number of ways. I look at each in turn and address the subordinate aims in each section, i.e., what factors:

- Affect whether students learn or not in a small-group setting?
- Enable students to learn effectively?
- Hinder students from effective learning?
- Is it possible to better manage the learning environment to promote learning?

### Learning ‘smart’, rather than learning it all

A key factor in coming from an outcomes-based approach where passing exams with top grades, is mastering how to pace learning for the development of understanding. This is tricky to overcome, as everything that students have done to gain a place at medical school has been based on attaining high grades. Within this, assessment plays a part and although Peninsula assessment has been set-up to ensure a frequent look at progress (Ricketts et al., 2010; Rodway-Dyer, 2010), students start with the notion that the AMK is there to be ‘passed’. It may be that this is not something that can be overcome and that life-long learning, in the academic sense, is something that each individual student comes to at their own pace. Clinical experience does appear to impact on understanding the concept and perhaps the programme already engenders an optimal approach to guiding students to this conclusion.

### Understanding the spiral curriculum

This theme ties in with learning smart. Students have been used to learning for an endpoint, not learning for life. Thus, learning outcomes have been short-term goals, rather than lifelong approaches. The PBL approach does appear to facilitate learning development in this respect, in that students are encouraged to link their learning. Three of the participant groups suggested that whilst they found the concept of the spiral curriculum frustrating at first – by Year 3 there was an acceptance that it worked, and they could see its value.

### Learning together

One of the most prominent factors in helping students to adapt their learning, seemed to be focused on educational background. Participants from schools that used a discussion-based and cooperative learning approach had a distinct advantage [e.g., Belinda (pair A) and Hannah (pair D)]. This is perhaps not surprising, particularly, as a school where this is not the case is likely to raise students for whom competition is a key driver, which appears to impinge on learning development. Freddie (pair C) did not express whether he came from a background of cooperative learning, but he seems to wholeheartedly accept it as a good way of learning. The small study group size meant that it is not possible to determine whether he is an exception or one of a larger sub-group, for whom, for whatever reason, collaborative learning is attractive from its first introduction. For the other participants in this study, collaboration appears to be a learned skill and thus, early exposure to a collaborative learning approach is more likely to prepare students for a programme where this is an expectation. Clearly, it is not possible or desirable to select students based on their school’s learning approach (particularly as it would appear this is a learnable skill). However, in terms of small group learning, the use of team activities for small groups early in the course might help students to see the advantage of learning more collaboratively. This is likely to work in conjunction with other issues that appear to hinder learning, such as understanding assessment, particularly in terms of progress rather than as a finite determinant of knowledge.

### Integrated learning

Students in Year 1, as school leavers, used to learning facts for exams, at first struggle with learning across disciplines and topics. This seems most clearly apparent in the learning of anatomy, where several of the participants comment that it is only possible to learn anatomy by rote, rather than in the context of other learning. Comments made by Annie (pair A) and Clare (pair B) suggest that they acknowledge that the lack of context is a factor, presumably this is a position that comes from reflection of their earlier learning methods, and it is when they start to consider their more effective learning in terms of their clinical exposure.

A key point of transition for the participants in the study appears to occur when they start to meet and have more in-depth conversations with patients when they progress to Year 3. This is highlighted by Freddie and Eashi (pair C), as they talk about how they have both considered the effects of psychosocial factors on their patients’ wellbeing. Again, this suggests that learning in context is tightly associated with experiential learning in practice. This is interesting from the point of view that students have early clinical exposure in Year 1 of their studies at Peninsula, yet in most cases, they do not appear to make the connection that weaving their learning into clinical context is advantageous until Year 3 of the programme. It seems that this transition is reliant on the development of skills in parallel before they can be combined and used simultaneously. It would be interesting to pursue this line of query to determine whether this is a rite of passage, or if experiential learning from the outset might lead to earlier integration of academic and experiential learning.

### Assessment and resources

The data presented here seems to suggest that the progress testing of Year 1 and 2 students distracts them from deep learning. Indeed, it is recognised by participants that student learning specifically for AMK has developed as a culture amongst Year 1 and 2 students, whereas by Year 3, there is an acceptance of its nature as a test of developing progress in clinical understanding. Thus, it might be surmised that the AMK is counterproductive in terms of learning development, in the first 2 years of the programme. However, another way of considering this could be that to understand how the AMK works, it is necessary to try to pass it in the first instance, this is particularly likely to be the case in students who have been top scorers in their exams to this point. It is difficult to envisage how the development of an earlier understanding of the test can be instilled. However, changing the stakes in terms of results may alter the way AMK is viewed, which may promote a better understanding of its aim. There have been numerous discussions around whether to make AMK formative in the first year, given the observations from this study, there is a chance that this would reduce the anxiety for students who would be able to see how they are progressing, without the threat of failure and might lead them to take a more relaxed view towards the test. On the other hand, for students who fail to take a formative test seriously, it could have the effect of causing the anxieties currently experienced in Year 1 to be pushed into Year 2, where the stakes would be higher. Another suggestion would be to allow collaboration between students in their first progress tests, which would generate an opportunity for developing group learning, alongside a development of understanding how the progress test works.

## Methodological review

The method used for this study demonstrated the value of conversation in eliciting student views on their developing learning. The BBC listening project has long since provided interesting insights into the shared, lived experiences of ordinary people and this is what prompted the approach used here (“Radio 4: The Listening Project”, 2016). The use of this method in collecting data from student participants does appear, at least to some extent, to ameliorate the relationship between students as participant and facilitator / tutor as researcher. The narratives collected provided a rich seam of data, which could be further analysed to study the relationships of students with learning in different ways, beyond the scope of this project, such as, how discussion about learning impacts on the understanding of learning and how separating learning into disciplines, e.g., anatomy in some aspects of an integrated curriculum.

A further observation, regarding this approach, points towards the value of conversation / talking together, thus introducing opportunities for students to discuss and reflect on their learning may lead to a better understanding of the overall process of development of learning skills over the first 2 years of the course, as was in evidence, particularly in the conversations of Alice and Belinda (pair A), where several instances of apparent change in their comprehension of how the learning activities facilitated their understanding of learning were apparent. Such an intervention would be innovative in curriculum development by facilitating a powerful tool in the armoury of personal and professional development in the early years of medicine.

In the analysis of the data, the use of the auto/biographical reflection to assert my position, proved helpful, as it enabled me to look at the conversational narrative from my own- alongside the participant- perspective. By acknowledging my own experience, it was possible to evaluate the participant experience in terms of both similarity and difference. In some cases, the conversational analysis cemented my own understanding, such as in terms of the spiral curriculum, where the concept struck me early on and helped me to understand how learning developed. At other times, the conversations, alongside my own position, helped my understanding to develop further, e.g., I became aware that the PBL process had for me been a key to understanding the iterative process of learning. However, I realised that this came from the experience of being able to look at PBL from a curriculum development point of view, rather than from that of the learner; and that from within the process, where the learning seems overwhelming, students may find it difficult to stand back and appreciate it in the same way.

## Limitations and further work

There are several limitations to this qualitative study. One of the major limitations was the number of students who volunteered to be participants. To develop a more detailed understanding of the commonalities between factors that help or hinder students in their early learning development in HE would require analysis of a greater number and variety of conversations between student pairs. However, it should be noted that there was a degree of convergence in terms of the narratives collected, which would suggest that the number required to achieve this may not be prohibitive (David and Sutton, 2011a). Additionally, I was not able to get the participants together as a focus group to discuss the findings from the conversations collected. This would have added a further dimension to the study in terms of discussing common enablers and barriers to learning development in the first years of study on the medical programme. The transferability of the results reported is limited as this Medical School is different from others in the UK (indeed all are to an extent, individual in their approaches). Furthermore, because of the study group size the results do not provide a complete generalisable set of data, although the individual issues raised are still valuable in the context of the participant group as a representative of the overall cohort.

Further work with similar programmes would be valuable. There are two ways that I would like to broaden the study, *viz* to look at whether the learning journeys of students at this medical school are comparable with students in similar and different (more traditional) schools and within this environment, how students from a widening participation background compare in terms of their adaptation to the programme. There is also scope to build on the data presented here regarding assessment in a study to determine whether the approach to the progress test in the first year could be developed to promote an earlier understanding of its purpose.

## Summary conclusion

The project on which this paper reports considers how student learning develops from Year 1 to Year 3 of a specific BMBS programme. My interest was sparked from an observation that some students found the transition from school to learning within a university setting on a programme where students must be self-guided, professional and learn through an integrated approach. From this work, I have developed an understanding of some of the drivers and developments that help students to make this transition *viz* learning what to learn, rather than trying to learn everything; which is generated from developing an understanding of the iterative nature of learning and facilitated by the spiral nature of this particular curriculum; understanding the nature of learning development through working as a group; learning that holistic learning in context makes understanding easier to achieve and that assessment drives learning in students in the earlier years, but as time progresses assessment is more a monitor of progress than an exam to be passed. My aims were to find out what:

- Affects whether students learn or not in a small-group setting?
- Enables students to learn effectively?
- Hinders students from effective learning?
- and
- Is it possible to better manage the learning environment to promote learning?

From the results presented here there are a number of issues: if students have been used to discussion in their school setting, they are more likely to adapt to this way of learning; group make-up in the earlier years may be a barrier to effective learning, but as time progresses and there is more exposure to clinical experiences, students begin to understand that it is up to them to work with the group and that it is important that they do this as a matter of good clinical working and practice. Learning skills must be developed to gain an understanding of how to maximise learning. It is no longer sufficient to pass exams as the overall assessment is of a wholesale approach to medicine, which in part includes some knowledge, but much more than that in terms of professionalism, team working and clinical capability. This is something that develops over time, but that could be improved by developing modes of assessment to better reflect the skills required – e.g., adapting progress testing in the earlier years to encourage group learning and integrated development of understanding. The evidence here suggests that learning is hindered when students do not adapt to the learning environment – earlier understanding of this may be facilitated by developing some of the areas described, but there may be to some extent a natural progression, which cannot be accelerated.

Whilst this represents a relatively small study group, and it is recognised that the number of participants in the project is not large enough to form any firm generalisations about the overall issues in development in learning here (David and Sutton, 2011a). It is also recognised that this study was an initial exploration of the factors that influence learning, where thick description was the aim, so a small number of participants was appropriate to achieve this. Furthermore, the transcribed conversations presented a rich seam of exploration and synthesis of aspects of learning, where the main themes coming through in each conversation were similar. Thus, the data can be assumed to reflect some of the key themes that help or hinder learning in the context of transition to study at this medical school. In considering the themes raised here against some of those raised incidentally in a previous audio diary project, some issues might reasonably be used to inform further research in this area.

The understanding of development of learning of the participants discussed here is of immense value in terms of the further development of medical curricula in this context. The findings from this project have been applied to the development of the curriculum to a broader remit of enquiry-based learning (formerly PBL). This has promoted a broader scope of cases to allow for a greater diversity of learning stimuli to promote critical thinking and development of learning approaches over the first 2 years of this BMBS programme.

## Data availability statement

Anonymised raw data supporting the conclusions of this article will be made available by the authors, without undue reservation.

## Ethics statement

The studies involving humans were approved by Plymouth University Faculty of Health and Human Sciences and Peninsula Schools of Medicine and Dentistry Student Ethics Committee. The studies were conducted in accordance with the local legislation and institutional requirements. The participants provided their written informed consent to participate in this study. Written informed consent was obtained from the individual(s) for the publication of any potentially identifiable images or data included in this article.

## Author contributions

KG was the sole author of this work contributing conception, design, collection of data, analysis, and authorship of the manuscript.
